# Dynamics and bifurcation analysis of a state-dependent impulsive SIS model

**DOI:** 10.1186/s13662-021-03436-3

**Published:** 2021-06-12

**Authors:** Jinyan Wang

**Affiliations:** School of Mathematics and Information Science, North Minzu University, Yinchuan, 750021 P.R. China

**Keywords:** SIS model, State-dependent impulsive control, Bifurcations, Impulsive periodic solutions

## Abstract

Recently, considering the susceptible population size-guided implementations of control measures, several modelling studies investigated the global dynamics and bifurcation phenomena of the state-dependent impulsive SIR models. In this study, we propose a state-dependent impulsive model based on the SIS model. We firstly recall the complicated dynamics of the ODE system with saturated treatment. Based on the dynamics of the ODE system, we firstly discuss the existence and the stability of the semi-trivial periodic solution. Then, based on the definition of the Poincaré map and its properties, we systematically investigate the bifurcations near the semi-trivial periodic solution with all the key control parameters; consequently, we prove the existence and stability of the positive periodic solutions.

## Introduction

The emerge and re-emergence of infectious diseases have always led to unpredictable epidemics and posed serious challenges to public health. In the past 200 years, at least seven waves of cholera, four new strains of influenza virus, tuberculosis, and HIV spread across the world and resulted in at least 100 million deaths [1]. Particularly in developed countries, infectious disease mortality disproportionately affects indigenous and disadvantaged minorities [2]. Since the outbreak of COVID-19 at the end of 2019, it has spread to over 200 countries with 163,212,543 confirmed cases and 3,383,979 deaths globally till May 18, 2021 [3]. Therefore, it is crucial to conduct an early prediction and give warning of the epidemics for guiding the development of control interventions [4] and to evaluate the efficacy of different strategies of control interventions in successfully controlling the spread of the disease [5–7].

As an attractive tool, mathematical models have been widely applied to understand the transmission mechanism of infectious diseases and to provide evidence for the effects of interventions in different groups of populations. During this COVID-19 pandemic, various mathematical models have been developed and further studied [8–10], which played a key role in controlling the spread of the virus [5, 11]. We note that, in terms of control strategies, the state-dependent feedback control was frequently used aiming at preventing the spread of infectious diseases [12–14]; consequently, abundant state-dependent impulsive models have been proposed [15–19]. Fractional calculus is a generalization of the standard integer calculus. It has become a significant field of investigation due to its immense opportunity and wide applications. A considerable number of articles have been concerned with reviewing the presence of solutions to fractional systems. Several researchers pioneered their attempts to extend the impulsive modelling methods to the fractional models [20–23] or the fractional integro-differential equations [24, 25], with various new analytic techniques developed and lots of interesting results obtained [26]. Gupta et al. in [20] investigated the existence and uniqueness of impulsive dynamical fractional systems with quadratic perturbation of second type subject to nonlocal boundary conditions. Kumar et al. in [22] explored the existence of solution of non-autonomous fractional differential equations with integral impulse condition by the measure of non-compactness (MNC), fixed point theorems, and *k*-set contraction. Ravichandran in [24] discussed the existence and uniqueness of solution to the integro-differential equations involving Atangana–Baleanu fractional derivatives.

Recently, several studies have introduced the susceptible population guided impulsive control into the SIR-type models and systematically investigated the dynamic behaviors [27–30]. In this study, we extend this modelling idea to the SIS-type model and investigate the rich dynamical behaviors and the bifurcations. The classical SIS-type model is given by

1.1 $$\begin{aligned} \textstyle\begin{cases} \frac{dS(t)}{dt}=A-dS-\beta \mathit{SI}+\mathit{vI}, \\ \frac{dI(t)}{dt}=\beta \mathit{SI}- (d+v )I, \end{cases}\displaystyle \end{aligned}$$

where *S* and *I* are the populations of susceptible and infectious, respectively. *A* is the recruitment rate of the susceptible populations, and *d* is the natural death rate. *β* is the property of transmission *per* contact, and the incidence rate is denoted by *βSI*. The recovery from the infected compartment is presented by *vI*. Furthermore, considering the continuous interventions, including the treatment and vaccination for controlling infectious diseases, system (1.1) becomes the following three-dimensional SIVS model:

1.2 $$ \textstyle\begin{cases} \frac{dS(t)}{dt}=A-dS-\beta \mathit{SI}+\mathit{vI}+\theta V+\frac{\mathit{cI}}{b+I}-\mathit{qS}, \\ \frac{dI(t)}{dt}=\beta \mathit{SI}- (d+v )I-\frac{\mathit{cI}}{b+I}, \\ \frac{\,dV(t)}{dt}=\mathit{qS}-\theta V-dV. \end{cases} $$

Here, the added compartment *V* denotes the vaccinated population. The recovery from the infected compartment with hospital treatment is represented by $\frac{\mathit{cI}}{b+I}$. Moreover, the susceptible population is vaccinated with rate *q*, and the vaccine protection wanes with rate *θ*. Then, we can extend model (1.2) by including the state-dependent control strategies and replacing the continuous vaccination with impulsive vaccination, and we obtain the following state-dependent impulsive model:

1.3 $$ \textstyle\begin{cases} \left . \textstyle\begin{array}{l} \frac{dS(t)}{dt}=A-dS-\beta \mathit{SI}+\mathit{vI}+\theta V+\frac{\mathit{cI}}{b+I}, \\ \frac{dI(t)}{dt}=\beta \mathit{SI}- (d+v )I-\frac{\mathit{cI}}{b+I}, \\ \frac{\,dV(t)}{dt}=-\theta V-dV, \end{array}\displaystyle \right \} S(t)< S_{T}, \\ \left . \textstyle\begin{array}{l} S(t^{+})= (1-q )S(t), \\ I(t^{+})=I(t), \\ V(t^{+})=V(t)+\mathit{qS}(t), \end{array}\displaystyle \right \} S(t)=S_{T}. \end{cases} $$

Here, $q\in (0,1)$ denotes the vaccination rate of the susceptible population.

## Preliminaries

Subject to the restriction $N(t)=S(t)+I(t)+V(t)$ for the ODE system of model (1.3), we have

$$ \frac{\mathit{dN}(t)}{dt}=A-dN(t), $$

which means that $N(t)$ will tend to $\frac{A}{d}$ as *t* approaches infinity. Without loss of generality, the ODE system in model (1.3) can be reduced to the following system:

2.1 $$ \textstyle\begin{cases} \frac{dS(t)}{dt}=A-dS-\beta \mathit{SI}+\mathit{vI}+\theta (\frac{A}{d}-S-I )+\frac{\mathit{cI}}{b+I}, \\ \frac{dI(t)}{dt}=\beta \mathit{SI}- (d+v )I-\frac{\mathit{cI}}{b+I}. \end{cases} $$

Consequently, the proposed state-dependent impulsive model (i.e. system (1.3)) is reduced to the following system:

2.2 $$ \textstyle\begin{cases} \left . \textstyle\begin{array}{l} \frac{dS(t)}{dt}=A-dS-\beta \mathit{SI}+\mathit{vI}+\theta (\frac{A}{d}-S-I )+\frac{\mathit{cI}}{b+I}, \\ \frac{dI(t)}{dt}=\beta \mathit{SI}- (d+v )I-\frac{\mathit{cI}}{b+I}, \end{array}\displaystyle \right \} S(t)< S_{T}, \\ \left . \textstyle\begin{array}{l} S(t^{+})= (1-q )S(t), \\ I(t^{+})=I(t), \end{array}\displaystyle \right \} S(t)=S_{T}. \end{cases} $$

We start with concluding the main dynamics of ODE system (2.1), while the detailed proof is similar to the existing study [31]. From biological considerations, we study (2.1) in the closed set

$$ D=\bigl\{ (S,I)|S,I\geq 0,S+I\leq A/d\bigr\} , $$

which is invariant set under nonnegative initial conditions.

Let the right-hand side of (2.1) be zero, we obtain that (2.1) has a disease-free equilibrium $E_{0}= (\frac{A}{d},0 )$. Using the notation in van den Driessche and Watmough [32], the reproduction number is given by

2.3 $$ R_{0}=\frac{\mathit{Ab}\beta }{d (c+b(d+v) )}. $$

Note that the characteristic equation of (2.1) at $E_{0}$ reads

2.4 $$ \bigl(\lambda + (d+\theta ) \bigr) \biggl(\lambda + \biggl(d+v+ \frac{c}{b} \biggr) (1-R_{0} ) \biggr)=0, $$

then it follows that all the eigenvalues of (2.4) have negative real parts if and only if $R_{0}<1$. Thus, we obtain the following.

### Proposition 1

*For model* (2.1), *the disease*-*free equilibrium* $E_{0}$ *is locally asymptotically stable if* $R_{0}<1$ *and unstable if* $R_{0}>1$.

In order to obtain the existence of endemic equilibria, we give the following quadratic equation from model (2.1) depending on the solutions of

$$ I^{2}+a_{1}I+a_{2}=0, $$

with

$$ a_{1}=b+\frac{d+v}{\beta }-\frac{A}{d},\qquad a_{2}= \frac{c+b(d+v)}{\beta }- \frac{\mathit{bA}}{d}. $$

For convenience, we denote

$$ I^{*}=\frac{-a_{1}+\sqrt{\Delta }}{2},\qquad S^{*}=\frac{A}{d}-I^{*}\quad \text{and}\quad I_{*}=\frac{-a_{1}-\sqrt{\Delta }}{2},\qquad S_{*}= \frac{A}{d}-I_{*}, $$

with $\Delta =a_{1}^{2}-4a_{2}$.

We further denote

$$ A_{1}=\mathit{bd}+\frac{d(d+v)}{\beta },\qquad \widehat{R}_{0}= \frac{4Abd\beta ^{2}}{4Abd\beta ^{2}+(d^{2}+dv+\mathit{bd}\beta -A\beta )^{2}}. $$

Then we get the result regarding the number of endemic equilibrium.

### Theorem 2

*For model* (2.1), *with* $A_{1}$ *and* $\widehat{R}_{0}$ *defined as above*, *we have*:

(1) *When* $R_{0}>1$ *or* $R_{0}=1$ *and* $A>A_{1}$, *there is a unique endemic equilibrium* $E^{*}$.

(2) *When* $R_{0}<1$ *and* $A\leq A_{1}$, *there is no endemic equilibrium*.

(3) *When* $1>R_{0}>\widehat{R}_{0}$ *and* $A>A_{1}$, *there are two endemic equilibria* $E^{*}$ *and* $E_{*}$.

(4) *When* $R_{0}=\widehat{R}_{0}$ *and* $A>A_{1}$, $E^{*}$ *and* $E_{*}$ *coincide into a unique endemic equilibrium of multiplicity* 2.

(5) *When* $R_{0}<\widehat{R}_{0}$ *and* $A>A_{1}$, *there is no endemic equilibrium*.

### Remark 1

When $A>A_{1}$, this theorem shows that there exists $\widehat{R}_{0}$ ($0<\widehat{R}_{0}<1$) such that the model has a unique endemic equilibrium for $R_{0}=\widehat{R}_{0}$, then the model has two endemic equilibria for $1>R_{0}>\widehat{R}_{0}$ and a unique endemic equilibrium for $R_{0}=1$. This situation corresponds to a backward bifurcation which occurs at $R_{0}=1$. There is also a forward transcritical bifurcation at $E_{0}$ (where $R_{0}=1$) if $A\leq A_{1}$.

When model (2.1) has no endemic equilibria, we get the global stability of the disease-free equilibrium $E_{0}$.

### Theorem 3

*If* $R_{0}<1$ *and* $A\leq A_{1}$ *or* $R_{0}<\widehat{R}_{0}$ *and* $A>A_{1}$ *hold*, *then the disease*-*free equilibrium* $E_{0}$ *of* (2.1) *is globally asymptotically stable*.

Next, we study the stability of the endemic equilibria. Denote $H(I)=\theta +d+q+\beta I-\frac{\mathit{cI}}{(b+I)^{2}}$, we get the following results.

### Theorem 4

*Suppose* $R_{0}>1$ *or* $1>R_{0}>\widehat{R}_{0}$ *and* $A>A_{1}$, *the endemic equilibrium* $E^{*}$ *of model* (2.1) *is a stable node or focus when* $H(I^{*})>0$; $E^{*}$ *is an unstable node or focus when* $H(I^{*})<0$; *and* $E^{*}$ *is a center of the linear system of* (2.1) *when* $H(I^{*})=0$.

To investigate the dynamical behaviors of system (2.2), we first briefly summarize the basic definitions and properties of the impulsive semi-dynamical systems. We consider the following generalized planer impulsive semi-dynamical system with state-dependent feedback control:

2.5 $$ \textstyle\begin{cases} \frac{\mathit{dx}}{dt}=P(x,y),\qquad \frac{\mathit{dy}}{dt}=Q(x,y),\quad (x,y)\notin \Phi , \\ \Delta x=a(x,y),\qquad \Delta y=b(x,y),\quad (x,y)\in \Phi , \end{cases} $$

where $(x,y)\in R_{+}^{2}=\{(x,y):x\geq 0,y\geq 0\}$, $\Delta x=x^{+}-x$, and $\Delta y=y^{+}-y$. Here, *P*, *Q*, *a*, *b* are continuous functions from $R_{+}^{2}$ to *R*, $\Phi \subset R_{+}^{2}$ is the impulsive set. For $(x,y)\in \Phi $, the impulsive function $\psi :R_{+}^{2}\rightarrow R_{+}^{2}$ is defined as

$$ \psi (x,y)=z^{=}\bigl(x+a(x,y),y+b(x,y)\bigr), $$

with $z^{=}(x^{+},y^{+})$ called an impulsive point of $z=(x,y)$. Then, based on the definitions in [12], we can define the impulsive semi-dynamical system and the order-*k* period solution. Particularly, the following analogue of Poincaré criterion in [33] determines the local stability of an order-*k* periodic solution.

### Lemma 5

*Let* $\phi (x,y)$ *be a sufficiently smooth function with* $\operatorname{grad} \phi (x,y)\neq 0$. *The order*-*k* *periodic solution* $(x,y)=(\xi (t),\eta (t))$ *with period* *T* *of* (2.5) *is orbitally asymptotically stable if the Floquet multiplier* $\mu _{2}$ *satisfies* $|\mu _{2}|<1$. *Here*,

$$ \mu _{2}=\prod_{k=1}^{q} \Delta _{k}\exp \biggl[ \int _{0}^{T} \biggl( \frac{\partial P}{\partial x} \bigl(\xi (t),\eta (t)\bigr)+ \frac{\partial Q}{\partial y}\bigl(\xi (t),\eta (t)\bigr) \biggr)\,dt \biggr], $$

*where*

$$ \Delta _{k}= \frac{P_{+} (\frac{\partial b}{\partial y}\frac{\partial \phi }{\partial x}-\frac{\partial b}{\partial x}\frac{\partial \phi }{\partial y}+\frac{\partial \phi }{\partial x} )+Q_{+} (\frac{\partial a}{\partial y}\frac{\partial \phi }{\partial y}-\frac{\partial a}{\partial y}\frac{\partial \phi }{\partial x}+\frac{\partial \phi }{\partial y} )}{P\frac{\partial \phi }{\partial x}+Q\frac{\partial \phi }{\partial y}}, $$

*and* *P*, *Q*, $\frac{\partial a}{\partial x}$, $\frac{\partial a}{\partial y}$, $\frac{\partial b}{\partial x}$, $\frac{\partial b}{\partial y}$, $\frac{\partial \phi }{\partial x}$, $\frac{\partial \phi }{\partial y}$ *are calculated at the point* $(\xi (\tau _{k}),\eta (\tau _{k}))$, *and* $P_{+}=P(\xi (\tau _{k}^{+}),\eta (\tau _{k}^{+}))$, $Q_{+}=Q(\xi (\tau _{k}^{+}), \eta (\tau _{k}^{+}))$ *with* $\tau _{k}(k\in N)$ *is the time of the* *kth jump*.

In order to study the bifurcations of the Poincaré map defined by system (2.5), we introduce the following lemmas [34].

### Lemma 6

(Transcritical bifurcation)

*Let* $G:U\times I\rightarrow R$ *define a one*-*parameter family of maps*, *where* *G* *is* $C^{r}$ *with* $r\geq 2$, *and* *U*, *I* *are open intervals of the real line containing* 0. *Assume*

$$ \begin{aligned} (1)\quad &G(0,\alpha )=0\quad \textit{for all }\alpha ;\qquad &&(2)\quad \frac{\partial G}{\partial x}(0,0)=1; \\ (3)\quad &\frac{\partial ^{2} G}{\partial x\partial \alpha }(0,0)>0;\qquad &&(4)\quad \frac{\partial ^{2} G}{\partial x^{2}}(0,0)>0. \end{aligned} $$

*Then there are* $\alpha _{1}<0<\alpha _{2}$ *and* $\epsilon >0$ *such that*:

(1) *If* $\alpha _{1}<\alpha <0$, *then* $G_{\alpha }$ *has two fixed points*, 0 *and* $x_{1\alpha }>0$ *in* (−*ϵ*, *ϵ*). *The origin is asymptotically stable*, *while the other fixed point is unstable*.

(2) *If* $0<\alpha <\alpha _{2}$, *then* $G_{\alpha }$ *has two fixed points*, 0 *and* $x_{1\alpha }<0$ *in* (−*ϵ*, *ϵ*). *The origin is unstable*, *while the other fixed point is asymptotically stable*.

Note that making the change of parameter $\alpha \rightarrow -\alpha $, we can handle the case $\frac{\partial ^{2}G(0,0)}{\partial x\partial \alpha }<0$.

### Lemma 7

(Supercritical pitchfork bifurcation)

*Let* $G:U\times I\rightarrow R$ *define a one*-*parameter family of maps*, *where* *G* *is* $C^{r}$ *with* $r\geq 3$, *and* *U*, *I* *are open intervals of the real line containing* 0. *Assume that*

$$ \begin{aligned} (1)&\quad G(0,\alpha )=0\quad \textit{for all } \alpha ;\qquad &&(2)\quad \frac{\partial G}{\partial x}(0,0)=1; \\ (3)&\quad frac{\partial ^{2} G} {\partial x\partial \alpha }(0,0)>0; \qquad &&(4)\quad \frac{\partial ^{2} G}{\partial x^{2}}(0,0)=0\quad \textit{and}\quad \frac{\partial ^{3} G}{\partial x^{3}}(0,0)< 0. \end{aligned} $$

*Then there are* $\alpha _{1}<0<\alpha _{2}$ *and* $\epsilon >0$ *such that*:

(1) *If* $\alpha _{1}<\alpha <0$, *then* $G_{\alpha }$ *has a unique fixed point*, $x=0$ *in* (−*ϵ*, *ϵ*), *which is asymptotically stable*.

(2) *If* $0<\alpha <\alpha _{2}$, *then* $G_{\alpha }$ *has three fixed points*, 0 *and* $x_{1\alpha }<0<x_{2\alpha }$ *in* (−*ϵ*, *ϵ*). *The origin is unstable*, *while the other two fixed points are asymptotically stable*.

Similarly, note that we can handle the case $\frac{\partial ^{2}G(0,0)}{\partial x\partial \alpha }<0$ by making the change of parameter $\alpha \rightarrow -\alpha $.

## Poincaré map and its properties

In this section, we first define the Poincaré map of system (2.2), and then we discuss its main properties. Before moving to the details, we firstly provide some intuitive basis on the Poincaré map. Poincaré used a cross section (called the Poincaré section) to transverse the trajectory of continuous motion. Then, according to the discrete motion of the intersection points of the trajectory to the cross section, we can simply judge the trend of the continuous motion. In the diagram on the Poincaré section, the succeed point where the trajectory crosses the Poincaré section can be regarded as a mapping of the point where the trajectory crosses the Poincaré section last time, which can be denoted by $x_{n+1}=f(x_{n})$, $n = 1, 2, 3,\ldots $ . This is actually the Poincaré map, and its function is to study the continuous motion through a simple discrete mapping. Based on the above interpretation, we can easily know that the fixed points of the Poincaré map actually reflect the periodic motion of the phase space.

We assume that $S_{T}<\frac{A}{d}$ holds true. Denote the two isolines of the system

3.1 $$ \textstyle\begin{cases} \frac{dS(t)}{dt}=A-dS-\beta \mathit{SI}+\mathit{vI}+\theta (\frac{A}{d}-S-I )+\frac{\mathit{cI}}{b+I}=F_{1}(S,I), \\ \frac{dI(t)}{dt}=\beta \mathit{SI}- (d+v )I-\frac{\mathit{cI}}{b+I}=F_{2}(S,I), \end{cases} $$

as follows:

$$ L_{1}:\dot{S}=F_{1}(S,I)=0,\qquad L_{2}: \dot{I}=F_{2}(S,I)=0, $$

and define two sections as follows:

$$ L_{3}:S_{S_{T}}=\bigl\{ (S,I)|S=S_{T},I\geq 0 \bigr\} ,\qquad L_{4}:S_{\mathit{qS}_{T}}=\bigl\{ (S,I)|S=(1-q)S_{T},I \geq 0\bigr\} . $$

According to the definitions in the last section, we have that the impulsive function $H(S,I)$ can be defined as

$$ H_{1}(S,I)=(1-q)S,\qquad H_{2}(S,I)=I. $$

Set the section $S_{\mathit{qS}_{T}}$ as a Poincaré section. Choosing an initial point $P_{k}^{+}=((1-q)S_{T},I_{k}^{+})$ on the Poincaré section, and the orbit starting from $P_{k}^{+}$ reaches $S_{S_{T}}$. We denote the intersection point as $P_{k+1}=(S_{T},I_{k+1})$, then the trajectory will jump to $P_{k+1}^{+}=((1-q)S_{T},I_{k+1})$ on the section $S_{\mathit{qS}_{T}}$. It follows from the existence and uniqueness of solutions that $I_{k+1}$ is identically determined by $I_{k}^{+}$. Therefore, we can define a function *g* with $g (I_{k}^{+} )=I_{k+1}$, and then the Poincaré map $\mathcal{P}_{1}$ can be defined as follows:

$$ \mathcal{P}_{1}:I_{k+1}^{+}=I_{k+1}=g \bigl(I_{k}^{+} \bigr)\doteq \mathcal{P}_{1} \bigl(q,A,S_{T},I_{k}^{+}\bigr). $$

Since the domain and range of the Poincaré map depends on the dynamics of system (3.1), we conclude the dynamics of system (3.1) by considering the following cases:

$(C_{1}) R_{0}<1$ and $A\leq A_{1} $ or $R_{0}< \widehat{R}_{0}$ and $A>A_{1}$;

$(C_{2}) R_{0}>1$ and $H(I^{*})>0$;

$(C_{3}) R_{0}>1$ and $H(I^{*})<0$;

$(C_{4}) 1>R_{0}>\widehat{R}_{0}$ and $A>A_{1}$.

Note that the phase trajectories of system (3.1) for case $(C_{3})$ or $(C_{4})$ show complexity, which leads to the complicated impulsive and phase sets of impulsive system (2.1). In what follows we discuss the definitions of the impulsive set and the phase set of system (2.2) for case $(C_{1})$ and case $(C_{2})$. Under case ($C_{1}$), the DFE $E_{0} (\frac{A}{d},0 )$ is globally asymptotically stable. It follows from the properties of the vector fields of system (3.1) that there exists an orbit $\Gamma _{1}$ tangent to $S_{\mathit{qS}_{T}}$ at the point $Q_{\mathit{qS}_{T}}= ((1-q)S_{T},I_{\mathit{qS}_{T}} )$ with $I_{\mathit{qS}_{T}}$ satisfying

3.2 $$ \mathit{BI}_{\mathit{qS}_{T}}^{2}+(D+ \mathit{Bb}+c)I_{\mathit{qS}_{T}}+\mathit{Db}=0, $$

with $B=v-\theta -\beta (1-q)S_{T}$, $D=(d+\theta ) (\frac{A}{d}-(1-q)S_{T} )>0$. Note that if $B\geq 0$, there is no positive solution of equation (3.2), while if $B<0$, there is a unique positive solution of (3.2):

$$ I_{qS_{T}}=\frac{-(D+Bb+c)-\sqrt{(D+Bb+c)^{2}-4BDb}}{2B}. $$

The intersection point of $\Gamma _{1}$ to $S_{S_{T}}$ is denoted by

$$ Q^{*}= \bigl(S_{T},I^{*} \bigr)= \bigl(S_{T},I \bigl(S_{T};(1-q)S_{T},I_{qS_{T}} \bigr) \bigr). $$

Then the impulsive set is

$$ M=\bigl\{ (S,I)|S=S_{T},0\leq I\leq I^{*}\bigr\} , $$

and the phase set *N* can be defined as follows:

$$ N=H(M)=\bigl\{ \bigl(S^{+},I^{+}\bigr)|S^{+}=(1-q)S_{T}, 0\leq I\leq I^{*}\bigr\} . $$

For case ($C_{2}$), the endemic equilibrium $E^{*}(S^{*},I^{*})$ is a stable focus or node. If $S_{T}< S^{*}$ holds, the impulsive set and the phase set of system (2.2) are similar to case ($C_{1}$). If $S_{T}>S^{*}$, we let

3.3 $$ B_{1}I_{S_{T}}^{2}+(D_{1}+B_{1}b+c)I_{S_{T}}+D_{1}b=0, $$

with $B=v-\theta -\beta S_{T}$, $D=(d+\theta ) (\frac{A}{d}-S_{T} )>0$. Note that if $B_{1}\geq 0$, there is no positive solution of equation (3.3), while if $B_{1}<0$, there is a unique positive solution of (3.3):

$$ I_{S_{T}}=\frac{-(D_{1}+B_{1}b+c)-\sqrt{(D_{1}+B_{1}b+c)^{2}-4B_{1}D_{1}b}}{2B_{1}}. $$

We assume $B_{1}<0$, then there exists an orbit $\Gamma _{2}$ tangent to the set $S_{S_{T}}$ at the point $Q_{S_{T}}= (S_{T},I_{S_{T}} )$. And we denote the intersection point of the orbit $\Gamma _{2}$ to the section $S_{\mathit{qS}_{T}}$ as $Q_{q}=((1-q)S_{T},I_{q})$ with $I(S_{T};(1-q)S_{T},I_{q})=I_{S_{T}}$. Any solution of model (2.2) with initial value ($S_{0}^{+}$, $I_{0}^{+}$) with $S_{0}^{+}=(1-q)S_{T}$ and $I_{0}^{+}\in (0,I_{q})$ will reach the section $S_{S_{T}}$ at finite time. Therefore, the impulsive set and the phase set of system can be defined as

$$ M=\bigl\{ (S,I)|S=S_{T},0\leq I\leq I_{S_{T}}\bigr\} $$

and

$$ N=H(M)=\bigl\{ \bigl(S^{+},I^{+}\bigr)|S^{+}=(1-q)S_{T}, 0\leq I\leq I_{S_{T}}\bigr\} , $$

respectively.

## Semi-trivial periodic solution

### Existence and stability of semi-trivial periodic solution

Let $I(t)=0$ for all $t\in (0,+\infty )$, system (2.2) becomes the following subsystem:

4.1 $$ \textstyle\begin{cases} \frac{dS(t)}{dt}=(d+\theta ) (\frac{A}{d}-S ),&S(t)< S_{T}, \\ S(t^{+})=(1-q)S(t),&S(t)=S_{T}. \end{cases} $$

Integrating the first equation of (4.1) with the initial conditions $S(0)=(1-q)S_{T}$, we have

$$ S(t)=\frac{A}{d}- \biggl(\frac{A}{d}-(1-q)S_{T} \biggr)\exp \bigl(-(d+ \theta )t\bigr). $$

Let $\frac{A}{d}- (\frac{A}{d}-(1-q)S_{T} )\exp (-(d+\theta )T)=S_{T}$, and solving it with respect to *T*, we get the period

$$ T=\frac{1}{d+\theta }\ln \frac{A-d(1-q)S_{T}}{A-dS_{T}}. $$

Therefore, system (2.2) has a semi-trivial periodic solution with period *T*, which is given as follows:

4.2 $$ \textstyle\begin{cases} \widehat{S}(t)=\frac{A}{d}- (\frac{A}{d}-(1-q)S_{T} ) \exp (-(d+\theta )(t-(k-1)T)), \\ \widehat{I}(t)=0,\quad (k-1)T< t\leq \mathit{kT}, k\in N. \end{cases} $$

Denote $(\widehat{S}(t),\widehat{I}(t) )=(\xi (t),0)$, then we discuss the stability of the semi-trivial periodic solution $(\xi (t),0)$. There are

$$ \begin{gathered} P(S,I)=A-dS-\beta \mathit{SI}+\mathit{vI}+\theta \biggl(\frac{A}{d}-S-I \biggr)+ \frac{\mathit{cI}}{b+I}, \\ Q(S,I)=\beta \mathit{SI}- (d+v )I-\frac{\mathit{cI}}{b+I}, \\ \widehat{\alpha }(S,I)=-\mathit{qS}(t),\qquad \widehat{\beta }(S,I)=0, \quad \phi (S,I)=S-S_{T}, \\ \bigl(\xi (T),\eta (T)\bigr)=(S_{T},0),\qquad \bigl(\xi \bigl(T^{+}\bigr),\eta \bigl(T^{+}\bigr)\bigr)= \bigl((1-q)S_{T},0\bigr). \end{gathered} $$

It is easy to calculate that

$$ \begin{gathered} \frac{\partial P}{\partial S}=-d-\theta -\beta I,\qquad \frac{\partial Q}{\partial I}=-(d+v)+\beta S-\frac{\mathit{bc}}{(b+I)^{2}}, \\ \frac{\partial \widehat{\alpha }}{\partial S}=-q,\qquad \frac{\partial \widehat{\alpha }}{\partial I}=0,\qquad \frac{\partial \widehat{\beta }}{\partial S}=0,\qquad \frac{\partial \widehat{\beta }}{\partial I}=0,\qquad \frac{\partial \phi }{\partial S}=1,\qquad \frac{\partial \phi }{\partial I}=0, \end{gathered} $$

and

$$\begin{aligned} \Delta _{1}&= \frac{P_{+} (\frac{\partial \widehat{\beta }}{\partial I}\frac{\partial \phi }{\partial S}-\frac{\partial \widehat{\beta }}{\partial S}\frac{\partial \phi }{\partial I}+\frac{\partial \phi }{\partial S} )+Q_{+} (\frac{\partial \widehat{\alpha }}{\partial S}\frac{\partial \phi }{\partial I}-\frac{\partial \widehat{\alpha }}{\partial I}\frac{\partial \phi }{\partial S}+\frac{\partial \phi }{\partial I} )}{P\frac{\partial \phi }{\partial S}+Q\frac{\partial \phi }{\partial I}} \\ &=\frac{P_{+}-\mathit{qQ}_{+}}{P}= \frac{P(\xi (T^{+}),\eta (T^{+}))}{P(\xi (T),\eta (T))} \\ &=\frac{A-d(1-q)S_{T}}{A-dS_{T}}. \end{aligned}$$

Moreover, there is

$$\begin{aligned} &\exp \biggl( \int _{0}^{T} \biggl(\frac{\partial P}{\partial S}\bigl( \xi (t), \eta (t)\bigr)+\frac{\partial Q}{\partial I}\bigl(\xi (t),\eta (t)\bigr) \biggr) \,dt \biggr) \\ &\quad =\exp \biggl( \int _{0}^{T} \biggl(-2d-\theta -v- \frac{c}{b}+ \beta \xi (t) \biggr)\,dt \biggr) \\ &\quad =\exp \biggl( \int _{0}^{T} \biggl(-2d-\theta -v- \frac{c}{b}+ \frac{\beta A}{d}-\frac{\beta (A-d(1-q)S_{T})\exp (-(d+\theta )t)}{d} \biggr)\,dt \biggr) \\ &\quad =\exp \biggl( \frac{-2d-\theta -v-\frac{c}{b}+ \frac{\beta A}{d}}{d+\theta }\ln \frac{A-d(1-q)S_{T}}{A-dS_{T}}- \frac{\beta \mathit{qS}_{T}}{d+\theta } \biggr) \\ &\quad = \biggl(\frac{A-d(1-q)S_{T}}{A-dS_{T}} \biggr)^{ \frac{-2d-\theta -v-\frac{c}{b}+ \frac{\beta A}{d}}{d+\theta }}\exp \biggl(- \frac{\beta \mathit{qS}_{T}}{d+\theta } \biggr). \end{aligned}$$

Thus, the Floquet multiplier $\mu _{2}$ can be calculated as

$$\begin{aligned} \mu _{2}&=\Delta _{1}\exp \biggl( \int _{0}^{T} \biggl( \frac{\partial P}{\partial S} \bigl(\xi (t),\eta (t)\bigr)+ \frac{\partial Q}{\partial I}\bigl(\xi (t),\eta (t)\bigr) \biggr)\,dt \biggr) \\ &= \biggl(\frac{A-d(1-q)S_{T}}{A-dS_{T}} \biggr)^{ \frac{-d-v-\frac{c}{b}+ \frac{\beta A}{d}}{d+\theta }}\exp \biggl(- \frac{\beta \mathit{qS}_{T}}{d+\theta } \biggr)>0. \end{aligned}$$

Because $0<1-q<1$, $\exp (-\frac{\beta \mathit{qS}_{T}}{d+\theta } )>0$, and $\frac{A-d(1-q)S_{T}}{A-dS_{T}}>1$, we have that if $R_{0}<1$, then there are $\frac{-d-v-\frac{c}{b}+\frac{\beta A}{d}}{d+\theta }<0$ and $0< (\frac{A-d(1-q)S_{T}}{A-dS_{T}} )^{ \frac{-d-v-\frac{c}{b}+ \frac{\beta A}{d}}{d+\theta }}<1$; if $R_{0}>1$, then there are $\frac{-d-v-\frac{c}{b}+\frac{\beta A}{d}}{d+\theta }>0$ and $(\frac{A-d(1-q)S_{T}}{A-dS_{T}} )^{ \frac{-d-v-\frac{c}{b}+ \frac{\beta A}{d}}{d+\theta }}>1$.

It follows from the definition of Poincaré map and the property of system (3.1) that the Poincaré map $\mathcal{P}_{1}$ is monotonically decreasing under case ($C_{1}$) on the whole domain of definition. Therefore, the semi-trivial periodic solution $(\xi (t),0)$ is globally attractive. Based on the above discussion, we have the following conclusion.

#### Theorem 8

*If* $R_{0}<1$, *then the semi*-*trivial periodic solution is orbitally asymptotically stable*. *Particularly*, *for case* ($C_{1}$), *the semi*-*trivial periodic solution of system* (2.2) *is globally stable*.

### Bifurcation of the semi-trivial periodic solution

Consider system (3.1) in the phase space, we have

$$ \frac{d I}{d S}=\frac{F_{2}(S,I)}{F_{1}(S,I)}\doteq h(S,I). $$

Given an initial point $(S_{0},I_{0} )$ on the Poincaré section with $S_{0}=(1-q)S_{T}$, $I_{0}\in (0,I_{q})$, we can solve *I* with respect to *S* as follows:

$$ I(S;S_{0},I_{0})=I_{0}+ \int _{(1-q)S_{T}}^{S}h\bigl(s,I\bigl(s;(1-q)S_{T},I_{0} \bigr)\bigr)\,ds \doteq I(S,I_{0}). $$

$\mathcal{P}_{1}$ can be also represented as

$$ \mathcal{P}_{1}(I_{0},\alpha )=g(I_{0}; \alpha )=I\bigl(S_{v};(1-q)S_{v},I_{0} \bigr), $$

with $I_{0}\in (0,I_{q})$ as the variable, $\alpha \in \Theta $ being the bifurcation parameter. For example, if we consider the bifurcation with respect to *q*, then *α* means *q*. For convenience, we denote

$$ \frac{\partial I(S_{v};(1-q)S_{v},I_{0})}{\partial I_{0}}= \frac{\partial g(I_{0};\alpha )}{\partial I_{0}}\doteq g'(I_{0}; \alpha ). $$

Through easy calculation, there are

$$\begin{aligned}& \frac{\partial I(S,I_{0})}{\partial I_{0}}=\exp \int _{(1-q)S_{T}}^{S} \frac{\partial h(s,I(s,I_{0}))}{\partial I}\,ds, \\& \frac{\partial ^{2} I(S,I_{0})}{\partial I_{0}^{2}}=\frac{\partial I(S,I_{0})}{\partial I_{0}} \int _{(1-q)S_{T}}^{S} \frac{\partial ^{2} h(s,I(s,I_{0}))}{\partial I^{2}} \frac{\partial I(s,I_{0})}{\partial I_{0}}\,ds, \\& \begin{aligned} \frac{\partial ^{3} I(S,I_{0})}{\partial I_{0}^{3}}={}&\frac{\partial ^{2} I(S,I_{0})}{\partial I_{0}^{2}} \int _{(1-q)S_{T}}^{S} \frac{\partial ^{2} h(s,I(s,I_{0}))}{\partial I^{2}} \frac{\partial I(s,I_{0})}{\partial I_{0}}\,ds \\ &{}+\frac{\partial I(S,I_{0})}{\partial I_{0}} \int _{(1-q)S_{T}}^{S} \biggl[\frac{\partial ^{3} h(s,I(s,I_{0}))}{\partial I^{3}} \biggl( \frac{\partial I(s,I_{0})}{\partial I_{0}} \biggr)^{2}\\ &{}+ \frac{\partial ^{2} h(s,I(s,I_{0}))}{\partial I^{2}} \frac{\partial ^{2} I(s,I_{0})}{\partial I_{0}^{2}} \biggr]\,ds. \end{aligned} \end{aligned}$$

Therefore, we have

$$\begin{aligned}& \frac{\partial \mathcal{P}_{1}}{\partial I_{0}}(0,\alpha )=g'(0; \alpha )=\mu_{2}>0, \\& \frac{\partial ^{2} \mathcal{P}_{1}}{\partial I_{0}^{2}}(0,\alpha )=g''(0; \alpha ), \\& \frac{\partial ^{3} \mathcal{P}_{1}}{\partial I_{0}^{3}}(0,\alpha )=g'''(0,\alpha ), \\& \frac{\partial ^{2} \mathcal{P}_{1}}{\partial I_{0}^{2}}(0,\alpha )=\frac{\partial \mu _{2}}{\partial \alpha }, \end{aligned}$$

with

$$\begin{aligned}& \begin{aligned} g'(0;\alpha )&=\frac{\partial I(S_{T},0)}{\partial I_{0}}=\exp \int _{(1-q)S_{T}}^{S_{T}} \frac{\partial h(s,I(s,0))}{dI}\,ds=\exp \int _{(1-q)S_{T}}^{S_{T}} \frac{\beta s-d-v-\frac{c}{b}}{ (\frac{A}{d}-s )(d+\theta )}\,ds \\ &= \biggl(\frac{A-d(1-q)S_{T}}{A-dS_{T}} \biggr)^{ \frac{-d-v-\frac{c}{b}+\frac{\beta A}{d}}{d+\theta }}\exp \biggl(- \frac{\beta \mathit{qS}_{T}}{d+\theta } \biggr)=\mu _{2}>0, \end{aligned} \\& \begin{aligned} g''(0;\alpha )&= \frac{\partial ^{2} I(S_{T},0)}{\partial I_{0}^{2}}=g'(0; \alpha ) \int _{(1-q)S_{T}}^{S_{T}} \frac{\partial ^{2} h(s,I(s,0))}{\partial I^{2}} \frac{\partial I(s,0)}{\partial I_{0}}\,ds \\ &=g'(0;\alpha ) \int _{(1-q)S_{T}}^{S_{T}}m(s) \frac{\partial I(s,0)}{\partial I_{0}}\,ds, \end{aligned} \\& \begin{aligned} g'''(0,\alpha )={}& \frac{\partial ^{3} I(S_{T},0)}{\partial I_{0}^{3}}\\ ={}&g'(0; \alpha ) \int _{(1-q)S_{T}}^{S_{T}} \biggl[n(s) \biggl( \frac{\partial I(s,0)}{\partial I_{0}} \biggr)^{2}+m(s) \frac{\partial ^{2} I(s,0)}{\partial I_{0}^{2}} \biggr] \,ds\\ &{}+g''(0;\alpha ) \int _{(1-q)S_{T}}^{S_{T}}m(s) \frac{\partial I(s,0)}{\partial I_{0}}\,ds \\ ={}&g'(0;\alpha ) \int _{(1-q)S_{T}}^{S_{T}} \biggl[n(s) \biggl( \frac{\partial I(s,0)}{\partial I_{0}} \biggr)^{2}+m(s) \frac{\partial I(s,0)}{\partial I_{0}} \biggl( \int _{(1-q)S_{T}}^{S_{T}}m(s) \frac{\partial I(s,0)}{\partial I_{0}} \biggr) \biggr]\,ds \\ &{}+g''(0;\alpha ) \int _{(1-q)S_{T}}^{S_{T}}m(s) \frac{\partial I(s,0)}{\partial I_{0}}\,ds, \end{aligned} \end{aligned}$$

where

$$\begin{aligned}& m(s)=\frac{\partial ^{2} h(s,I(s,0))}{\partial I^{2}}= \frac{\frac{2c}{b^{2}} (\frac{A}{d}-s ) (d+\theta )-2 (\beta s-d-v-\frac{c}{b} ) (-\beta s+v-\theta +\frac{c}{b} ) }{ (\frac{A}{d}-s )^{2} (d+\theta )^{2}}, \\& \begin{aligned} n(s)&=\frac{\partial ^{3} h(s,I(s,0))}{\partial I^{3}}\\ &= \frac{\frac{6c}{b^{2}} (\frac{A}{d}-s ) (d+\theta ) (2\beta s-2v-d+\theta -\frac{2c}{b} )-\frac{6c}{b^{3}} (\frac{A}{d}-s )^{2} (d+\theta )^{2}+6 (\beta s-d-v-\frac{c}{b} )^{2} (\beta s-d-v-\frac{c}{b} ) }{ (\frac{A}{d}-s )^{3} (d+\theta )^{3}}, \end{aligned} \\& \frac{\partial I(s,0)}{\partial I_{0}}= \frac{\partial I(S_{T},0)}{\partial I_{0}}= \biggl( \frac{A-d(1-q)S_{T}}{A-\,ds} \biggr)^{ \frac{-d-v-\frac{c}{b}+\frac{\beta A}{d}}{d+\theta }}\exp \biggl(- \frac{\beta (s-(1-q)S_{T} )}{d+\theta } \biggr), \\& \frac{\partial ^{2} I(s,0)}{\partial I_{0}^{2}}= \frac{\partial I(s,0)}{\partial I_{0}} \int _{(1-q)S_{T}}^{s} m(s) \frac{\partial I(s,0)}{\partial I_{0}}\,ds. \end{aligned}$$

#### Bifurcations with respect to *q*

Firstly, we consider the existence of $q^{*}$ such that $\mu _{2}|_{q=q^{*}}=1$. Taking the derivative of $\mu _{2}$ with respect to *q* yields

$$ \frac{\partial \mu _{2}}{\partial q}= \frac{\mu _{2}\,dS_{T} (\beta (1-q)S_{T}-d-v-\frac{c}{b} )}{(d+\theta )(A-d(1-q)S_{T})}. $$

Definitely, there is $\frac{\mu _{2}\,dS_{T}}{(d+\theta )(A-d(1-q)S_{T})}>0$, thus the sign of $\frac{\partial \mu _{2}}{\partial q}$ is determined by $\beta (1-q)S_{T}-d-v-\frac{c}{b}$. If $\frac{b(b+v)+c}{b\beta }< S_{T}<\frac{A}{d}$, there is $\widetilde{q}=1-\frac{b(b+v)+c}{b\beta S_{T}}\in (0,1)$, thus we have $\frac{\partial \mu }{\partial q}=0$ for $q=\widetilde{q}$, $\frac{\partial \mu }{\partial q}<0$ for $q>\widetilde{q}$, and $\frac{\partial \mu }{\partial q}>0$ for $q<\widetilde{q}$. This means that $\mu _{2}(q)$ is decreasing in the interval $(\widetilde{q},1)$ and increasing in the interval $(0,\widetilde{q})$. Furthermore, we have

$$ \mu _{2}|_{q=0}=1,\qquad \mu _{2}|_{q=1}= \biggl(\frac{A}{A-dS_{T}} \biggr)^{ \frac{-d-v-\frac{c}{b}+\frac{\beta A}{d}}{d+\theta }}\exp \biggl(- \frac{\beta S_{T}}{d+\theta } \biggr)>0, $$

with

$$ \biggl(\frac{A}{A-dS_{T}} \biggr)^{ \frac{-d-v-\frac{c}{b}+\frac{\beta A}{d}}{d+\theta }}>1. $$

Therefore, we have that

(1) If $\mu _{2}|_{q=1}\geq 1$, then there is no $q^{*}\in (0,1)$ satisfying $\mu _{2}|_{q=q^{*}}=1$.

(2) If $\mu _{2}|_{q=1}<1$, then there is $q^{*}=q_{1}^{*}\in (\widetilde{q},1)$ such that $\mu _{2}|_{q=q_{1}^{*}}=1$. And, it is easy to verify that $\frac{\partial ^{2}\mathcal{P}_{1}}{\partial I_{0}\partial q}(0,q_{1}^{*})<0$ holds true.

On the other hand, if $0< S_{T}<\frac{b(b+v)+c}{b\beta }$, then $\mu _{2}(q)$ is decreasing in the interval $(0,1)$. Thus, there is no $q^{*}\in (0,1)$ satisfying $\mu _{2}|_{q=q^{*}}=1$. Therefore, we have the following proposition.

##### Proposition 9

*If* $R_{0}>1$, $\frac{b(b+v)+c}{b\beta }< S_{T}<\frac{A}{d}$, *and* $\mu _{2}|_{q=1}\geq 1$ *hold true*, *then the semi*-*trivial periodic solution* $(\xi (t),0)$ *is unstable for* $q\in (0,1)$; *If* $R_{0}>1$, $\frac{b(b+v)+c}{b\beta }< S_{T}<\frac{A}{d}$, *and* $\mu _{2}|_{q=1}<1$ *hold true*, *then the semi*-*trivial periodic solution* $(\xi (t),0)$ *is orbitally asymptotically stable for* $q\in (q_{1}^{*},1)$ *and unstable for* $q\in (0,q_{1}^{*})$; *If* $R_{0}>1$ *and* $0< S_{T}<\frac{b(b+v)+c}{b\beta }$ *hold true*, *then the semi*-*trivial periodic solution* $(\xi (t),0)$ *is orbitally asymptotically stable for* $q\in (0,1)$.

In what follows, we consider the bifurcations with respect to *q* under the conditions of the existence of $q^{*}$. It is easy to see that there is $\mathcal{P}_{1}(0,q)=0$ for all $q\in (0,1)$, and

$$ \frac{\partial \mathcal{P}_{1}}{\partial I_{0}}\bigl(0,q_{1}^{*}\bigr)=1,\qquad \frac{\partial ^{2} \mathcal{P}_{1}}{\partial I_{0}\partial q}\bigl(0,q_{1}^{*}\bigr)< 0. $$

Moreover, we have

4.3 $$ g''\bigl(0;q_{1}^{*} \bigr)=g'\bigl(0;q_{1}^{*}\bigr) \int _{(1-q_{1}^{*})S_{T}}^{S_{T}}m(s) \frac{\partial I(s,0)}{\partial I_{0}}\,ds. $$

As a result of the indeterminacy of the sign of $m(s)$ in the interval $s\in ((1-q_{1}^{*})S_{T},S_{T})$, the sign of $g''(0;q_{1}^{*})$ is undetermined. If we assume $g''(0;q_{1}^{*})>0$, then we have

$$ \frac{\partial ^{2} \mathcal{P}_{1}}{\partial I_{0}^{2}}\bigl(0,q_{1}^{*} \bigr)=g''\bigl(0;q_{1}^{*} \bigr)>0. $$

Based on the above discussion, we have the following conclusion.

##### Theorem 10

*If* $R_{0}>1$, $\frac{b(b+v)+c}{b\beta }< S_{T}<\frac{A}{d}$, *and* $\mu _{2}|_{q=1}<1$ *hold true*, *then the Poincaré map* $\mathcal{P}_{1}(I_{0},q)$ *undergoes a transcritical bifurcation at* $p_{1}^{*}$. *Further*, *an unstable positive fixed point appears when* *q* *goes through* $q=q_{1}^{*}$ *from left to right*. *Correspondingly*, *system* (2.2) *has an unstable positive periodic solution if* $p\in (p_{1}^{*},p_{1}^{*}+\delta )$ *with* $\delta >0$.

##### Theorem 11

*If* $R_{0}>1$, $\frac{b(b+v)+c}{b\beta }< S_{T}<\frac{A}{d}$, $\mu _{2}|_{q=1}<1$, $g''(0;q_{1}^{*})=0$, *and* $g'''(0;q_{1}^{*})>0$ *hold true*, *then the Poincaré map* $\mathcal{P}_{1}(I_{0},q)$ *undergoes a supercritical pitchfork bifurcation at* $p_{1}^{*}$. *Therefore*, *an unstable positive fixed point appears when* *q* *goes through* $q=q_{1}^{*}$ *from left to right*. *Correspondingly*, *system* (2.2) *has an unstable positive periodic solution if* $p\in (p_{1}^{*},p_{1}^{*}+\delta )$ *with* $\delta >0$.

#### Bifurcation with respect to $S_{T}$

Similar to the case for *q*, we first analyze the existence of $S_{T}^{*}$ such that $\mu _{2}|_{S_{T}=S_{T}^{*}}=1$. Taking the derivative of $\mu _{2}$ with respect to $S_{T}$ yields

$$ \frac{\partial \mu _{2}}{\partial S_{T}}=\frac{q\mu _{2}}{d+\theta }f(S_{T}) $$

with

$$ f(S_{T})= \frac{\mathit{dA} (-d-v-\frac{c}{b}+\frac{\beta A}{d} )}{(A-d S_{T})(A-d(1-q)S_{T})}- \beta . $$

The roots of the equation $f(S_{T})=0$, denoted by $\overline{S}_{T}$, satisfy the following equation:

4.4 $$ \beta d(1-q)\overline{S}_{T}^{2}+\beta A(q-2)\overline{S}_{T}+A \biggl(d+v+ \frac{c}{b} \biggr)=0. $$

Let

$$\begin{aligned} \Delta =&\beta ^{2}A^{2}(q-2)^{2}+4\beta \mathit{Ad}(1-q) \biggl(d+v+ \frac{c}{b} \biggr) \\ &{}\times\beta ^{2}A^{2} \biggl((q-2)^{2}- \frac{4(1-q)}{R_{0}} \biggr). \end{aligned}$$

Thus, $\Delta >0$ is equivalent to $R_{0}>\frac{4(1-q)}{(q-2)^{2}}$. Denote $K(q)=\frac{4(1-q)}{(q-2)^{2}}$. There are $K(0)=1$ and $K'(q)=\frac{4q}{(q-2)^{3}}<0$. Therefore, we have that $K(q)<1$ holds for all $q\in (0,1)$. This means that $\Delta >0$ when $R_{0}>1$, hence equation (4.4) has two roots, denoted by

$$\begin{aligned}& \overline{S}_{T_{1}}= \frac{-\frac{A}{d}(q-2)-\frac{A}{d}\sqrt{(q-2)^{2}-\frac{4(1-q)}{R_{0}}}}{2(1-q)}, \\& \overline{S}_{T_{2}}= \frac{-\frac{A}{d}(q-2)+\frac{A}{d}\sqrt{(q-2)^{2}-\frac{4(1-q)}{R_{0}}}}{2(1-q)}. \end{aligned}$$

We can verify that $0<\overline{S}_{T_{1}}<\frac{A}{d}<\overline{S}_{T_{2}}$. Thus, $\mu _{2}$ is decreasing when $S_{T}\in (0,\overline{S}_{T_{1}})$ and increasing when $S_{T}\in (\overline{S}_{T_{1}},\frac{A}{d})$.

Furthermore, when $R_{0}>1$, there are

$$ \mu _{2}|_{S_{T}=0}=1,\qquad \mu _{2}|_{S_{T}=\overline{S}_{T_{1}}}< 1, \qquad \mu _{2}|_{S_{T}\rightarrow \frac{A}{d}^{-}}=+\infty . $$

Therefore, there is a unique $S_{T}^{*}\in (\overline{S}_{T_{1}},\frac{A}{d})$ such that $\mu _{2}|_{S_{T}=\overline{S}_{T_{1}}}=1$ with $S_{T}^{*}$ satisfying

$$ \biggl(\frac{A-d(1-q)S_{T}^{*}}{A-dS_{T}^{*}} \biggr)^{ \frac{-d-v-\frac{c}{b}+ \frac{\beta A}{d}}{d+\theta }}\exp \biggl(- \frac{\beta \mathit{qS}_{T}^{*}}{d+\theta } \biggr)=1. $$

Based on the above discussion, we can conclude the following.

##### Proposition 12

*If* $R_{0}>1$, *there exists unique* $S_{T}^{*}\in (\overline{S}_{T_{1}},\frac{A}{d})$ *such that* $\mu _{2}|_{S_{T}=S_{T}^{*}}=1$. *Then the semi*-*trivial periodic solution* $(\xi (t),0)$ *is orbitally asymptotically stable for* $S_{T}\in (0,S_{T}^{*})$ *and unstable for* $S_{T}\in (S_{T}^{*},\frac{A}{d})$.

In what follows, we investigate the bifurcations of the semi-trivial periodic solution at $S_{T}^{*}$. We can easily verify that $\mathcal{P}_{1}(0,S_{T})=0$ holds true for all $S_{T}\in (0,\frac{A}{d})$, and

$$ \frac{\partial \mathcal{P}_{1}}{\partial I_{0}}\bigl(0,S_{T}^{*}\bigr)=1,\qquad \frac{\partial ^{2} \mathcal{P}_{1}}{\partial I_{0}\partial S_{T}}\bigl(0,S_{T}^{*}\bigr)>0. $$

Moreover, we have

4.5 $$ g''\bigl(0;S_{T}^{*} \bigr)=g'\bigl(0;S_{T}^{*}\bigr) \int _{(1-q)S_{T}^{*}}^{S_{T}^{*}}m(s) \frac{\partial I(s,0)}{\partial I_{0}}\,ds. $$

As a result of the indeterminacy of the sign of $m(s)$ in the interval $s\in ((1-q)S_{T}^{*},S_{T}^{*})$, the sign of $g''(0;S_{T}^{*})$ is undetermined. If we assume $g''(0;S_{T}^{*})>0$, then we have

$$ \frac{\partial ^{2} \mathcal{P}_{1}}{\partial I_{0}^{2}}\bigl(0,S_{T}^{*} \bigr)=g''\bigl(0;S_{T}^{*} \bigr)>0. $$

Based on the above discussion, we have the following conclusion.

##### Theorem 13

*If* $R_{0}>1$ *and* $g''(0;S_{T}^{*})>0$ *hold true*, *then the Poincaré map* $\mathcal{P}_{1}(I_{0},S_{T})$ *undergoes a transcritical bifurcation at* $S_{T}=S_{T}^{*}$. *Further*, *an unstable positive fixed point appears when* $S_{T}$ *goes through* $S_{T}=S_{T}^{*}$ *from right to left*. *Correspondingly*, *system* (2.2) *has an unstable positive periodic solution if* $S_{T}\in (S_{T}^{*}-\delta ,S_{T}^{*})$ *with* $\delta >0$.

##### Theorem 14

*If* $R_{0}>1$, $g''(0;S_{T}^{*})=0$ *and* $g'''(0;S_{T}^{*})>0$ *hold true*, *then the Poincaré map* $\mathcal{P}_{1}(I_{0},S_{T})$ *undergoes a supercritical pitchfork bifurcation at* $S_{T}=S_{T}^{*}$. *Therefore*, *an unstable positive fixed point appears when* $S_{T}$ *goes through* $S_{T}=S_{T}^{*}$ *from right to left*. *Correspondingly*, *system* (2.2) *has an unstable positive periodic solution if* $S_{T}\in (S_{T}^{*}-\delta ,S_{T}^{*})$ *with* $\delta >0$.

### Bifurcation with respect to *A*

Taking $\mu _{2}$ as a function of *A*, we have

$$ \mu _{2}(A)= \biggl(\frac{A-d(1-q)S_{T}}{A-dS_{T}} \biggr)^{ \frac{-d-v-\frac{c}{b}+\frac{\beta A}{d}}{d+\theta }} \exp \biggl( \frac{-\beta \mathit{qS}_{T}}{d+\theta } \biggr). $$

It is continuously differentiable when $A\in (dS_{T},+\infty )$, and there are

4.6 $$ \lim_{A\rightarrow \,dS_{T}^{+}}\mu _{2}(A)=+\infty ,\qquad \lim_{A \rightarrow +\infty }\mu _{2}(A)=\exp \biggl(- \frac{\beta \mathit{qS}_{T}}{d+\theta } \biggr)< 1. $$

Taking the derivative of $\mu _{2}$ with respect to *A* yields

$$ \frac{\partial \mu _{2}}{\partial A}=\frac{\beta \mu _{2}}{d+\theta }*B_{1}(A) $$

with

$$ B_{1}(A)=\ln \frac{A-d(1-q)S_{T}}{A-dS_{T}}- \frac{ (\frac{-d-v-\frac{c}{b}}{\beta }+\frac{A}{d} )\mathit{dqS}_{T}}{(A-d(1-q)S_{T})(A-dS_{T})}. $$

Then, taking the derivative of $B_{1}$ with respect to *A*, we have

$$ \frac{\partial B_{1}(A)}{\partial A}= \frac{\mathit{qS}_{T}}{(A-dS_{T})^{2}(A-d(1-q)S_{T})^{2}}*B_{2}(A), $$

with

$$\begin{aligned} B_{2}(A) =&(1-d)A^{2}+ \biggl(d^{2}(2-q)S_{T}+2d \biggl(d+v+\frac{c}{b} \biggr) \biggr)A \\ &{}+d^{2}S_{T} \biggl(\frac{(2-q) (d+v+\frac{c}{b} )}{\beta }-(1+d) (1-q)S_{T} \biggr). \end{aligned}$$

If $\frac{(2-q) (d+v+\frac{c}{b} )}{\beta }-(1+d)(1-q)S_{T} \geq 0$ i.e. $S_{T}\leq \frac{(2-q) (d+v+\frac{c}{b} )}{\beta (1+d)(1-q)}$. Under this case, $B_{2}(A)>0$ holds for $A\in (dS_{T},+\infty )$. Furthermore, there is $\lim_{A\rightarrow +\infty }B_{1}(A)=0$, hence $B_{1}(A)<0$ for $A\in (dS_{T},+\infty )$, which means that $\frac{\partial \mu _{2}}{\partial A}<0$ for $A\in (dS_{T},+\infty )$. Combining with (4.6), we have that there exists unique $A^{*}\in (dS_{T},+\infty )$ satisfying $\mu _{2}(A^{*})=1$ with $\frac{\partial \mu _{2}(A^{*})}{\partial A}<0$.

If $\frac{(2-q) (d+v+\frac{c}{b} )}{\beta }-(1+d)(1-q)S_{T}<0$ i.e. $S_{T}>\frac{(2-q) (d+v+\frac{c}{b} )}{\beta (1+d)(1-q)}$. Under this scenario, there exists one and only one positive $A_{1}$ such that $B_{2}(A_{1})=0$. If $A_{1}\leq \,dS_{T}$, then the situation is similar to $S_{T}\leq \frac{(2-q) (d+v+\frac{c}{b} )}{\beta (1+d)(1-q)}$ discussed above. If $A_{1}>dS_{T}$, when $A\in (dS_{T},A_{1})$, $B_{2}(A)<0$, hence $B_{1}(A)$ is decreasing. Similarly, we get that $B_{1}(A)$ is increasing when $A\in (A_{1},+\infty )$. Further, $\lim_{A\rightarrow +\infty }B_{1}(A)=0$, thus $B_{1}(A)<0$ holds for $A\in (A_{1},+\infty )$. If we also assume that $B_{1}(A)<0$ for $A\in (dS_{T},A_{1})$, then there is $\frac{\partial \mu _{2}}{\partial A}<0$ for $A\in (dS_{T},+\infty )$. Therefore, there is unique $A^{*}\in (dS_{T},+\infty )$ satisfying $\mu _{2}(A^{*})=1$ with $\frac{\partial \mu _{2}(A^{*})}{\partial A}<0$. On the other hand, if we assume that there is $\widetilde{A}\in (dS_{T},A_{1})$ such that $B_{1}(\widetilde{A})=0$, then $\mu _{2}$ is increasing for $A\in (dS_{T},\widetilde{A})$, and $\mu _{2}$ is decreasing for $A\in (\widetilde{A},+\infty )$. Therefore, there is also unique $A^{*}\in (\widetilde{A},+\infty )$ satisfying $\mu _{2}(A^{*})=1$ with $\frac{\partial \mu _{2}(A^{*})}{\partial A}<0$. Therefore, we have the following conclusion.

#### Proposition 15

*If* $R_{0}>1$ *holds*, *then there exists unique* $A^{*}>dS_{T}$ *satisfying* $\mu _{2}(A^{*})=1$ *with* $\frac{\partial \mu _{2}(A^{*})}{\partial A}<0$. *And the semi*-*trivial periodic solution* $(\xi (t),0)$ *is orbitally asymptotically stable when* $A\in (A^{*},+\infty )$ *and unstable when* $A\in (dS_{T},A^{*})$.

In what follows, we consider the bifurcations of the semi-trivial periodic solution at $A^{*}$. Similarly, we can easily verify that $\mathcal{P}_{1}(0,A)=0$ holds for all $A\in (dS_{T},+\infty )$, and $\frac{\partial \mathcal{P}_{1}}{\partial I_{0}}(0,A^{*})=1$, $\frac{\partial ^{2} \mathcal{P}_{1}}{\partial I_{0}\partial A}(0,A^{*})<0$. Moreover, we have

4.7 $$ \frac{\partial ^{2} \mathcal{P}_{1}}{\partial I_{0}^{2}}\bigl(0,A^{*}\bigr)=g'' \bigl(0;A^{*}\bigr). $$

Similarly, the sign of $g''(0;A^{*})$ is undetermined. If we assume $g''(0;A^{*})>0$, we have $\frac{\partial ^{2} \mathcal{P}_{1}}{\partial I_{0}^{2}}(0,A^{*})>0$, then there appears an unstable fixed point of the Poincaré map $\mathcal{P}_{1}$ when *A* passes through $A^{*}$ from left to right.

Based on the above discussion, we have the following conclusion.

#### Theorem 16

*If* $R_{0}>1$ *and* $g''(0;A^{*})>0$ *hold*, *then the Poincaré map* $\mathcal{P}_{1}(I_{0},A)$ *undergoes a transcritical bifurcation at* $A=A^{*}$. *Further*, *an unstable positive fixed point appears when* *A* *passes through* $A=A^{*}$ *from left to right*. *Correspondingly*, *system* (2.2) *has an unstable positive periodic solution if* $A\in (A^{*},A^{*}+\delta )$ *with* $\delta >0$.

#### Theorem 17

*If* $R_{0}>1$, $g''(0;A^{*})=0$, *and* $g'''(0;A^{*})>0$ *hold true*, *then the Poincaré map* $\mathcal{P}_{1}(I_{0},A)$ *undergoes a supercritical pitchfork bifurcation at* $A=A^{*}$. *Therefore*, *an unstable positive fixed point appears when* *A* *goes through* $A=A^{*}$ *from left to right*. *Correspondingly*, *system* (2.2) *has an unstable positive periodic solution if* $A\in (A^{*},A^{*}+\delta )$ *with* $\delta >0$.

**Figure 1:**
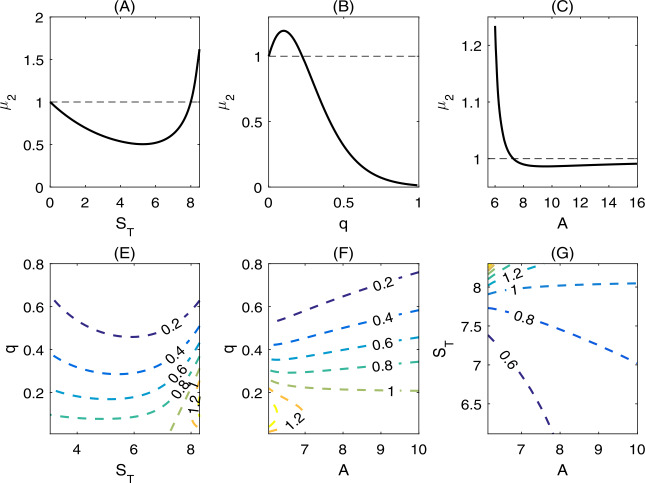
(A)-(C) One parameter bifurcation with respect to $S_{T}$, *q*, and *A*, respectively. (E)–(G) Counter plots of $\mu _{2}$. The baseline values of all the parameters are fixed as follows: $\beta =1$, $A=7$, $d=0.7$, $v=4$, $c=2.5$, $\theta =0.1$, $b=1$, *q*=, $0.22, S_{T}=8$

**Figure 2:**
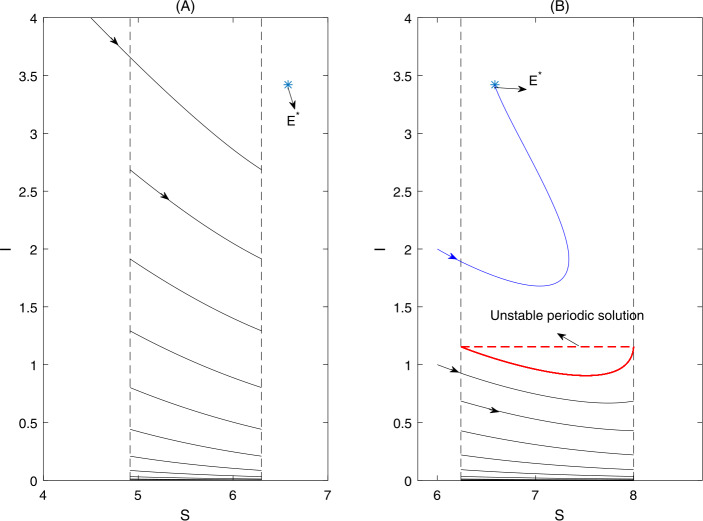
(A) The semi-trivial periodic solution is globally stable. (B) There exists an unstable positive order-1 periodic solution, and the semi-trivial periodic solution and the positive equilibrium $E^{*}$ are bistable. The other parameters are fixed as follows: $\beta =0.8$, $A=7$, $d=0.7$, $v=4$, $c=2.5$, $\theta =0.1$, $b=1$, $q=,0.22$

By choosing $S_{T}$, *q*, *A* as the bifurcation parameters and fixing all the other parameters, we numerically verified the existence of the critical bifurcation point with respect to these three parameters in Fig. 1. In Fig. 2(A), we showed that the semi-trivial periodic solution is globally stable when we choose $S_{T}=6.3$. As $S_{T}$ increases to 8, then an unstable periodic solution appears, the semi-trivial periodic solution and the positive equilibrium $E^{*}$ are bistable, as shown in Fig. 2(B).

## Conclusion and discussion

Recently, several studies pioneered the attempt to include the susceptible population-guided interventions for controlling infectious diseases into the SIR systems [27–30]. As highlighted in these studies, with this kind of control strategies, it is possible to define the control reproduction number for the impulsive system compared with the infected-population induced control interventions. In this study, we extended the SIS model by including the control strategy i.e. susceptible population-guided impulsive control, and systematically studied its dynamics and bifurcations.

We started with recalling the dynamic behavior of the ODE system. By defining the Poincaré map of the proposed model and presenting the proof of its properties, we explored the existence and stability of the semi-trivial periodic solution. We found a threshold parameter, which can be defined as the control reproduction number, determining the stability of the semi-trivial periodic solution. In detail, it is locally stable when the control reproduction number is less than 1 and unstable when the reproduction number exceeds the threshold value 1.

Furthermore, we investigated the bifurcations near the semi-trivial periodic solution considering the key parameters, including the constant recruitment rate *A*, the threshold level of the susceptible population $S_{T}$, and the pulse vaccination rate *q*. We proved that as the bifurcation parameters vary, the system can undergo the transcritical or pitchfork bifurcation near the semi-trivial periodic solution; consequently, the semi-trivial periodic solution loses its stability, while an unstable positive periodic solution appears. It is also interesting to summarize the biological implications by this impulsive SIS model and its dynamic behaviors and bifurcations. Through the bifurcation analysis with respect to $S_{T}$, we obtained a critical value of the threshold to guarantee the disease-free periodic solution to be stable. This means that by choosing a proper threshold of the susceptible population size, this kind of control strategy can indeed help to eliminate the disease. The bifurcations with respect to other parameters can actually reflect similar implications, like choosing a proper vaccination rate. On the other hand, the bistability of the positive equilibrium and the semi-trivial periodic solution indicate that the outcomes under this state-dependent control strategy depend on the initial conditions of the susceptible population and the infected population, hence a personalized strategy is recommended. Our model does not cover spatio-temporal delay, thus considering the effect of spatio-temporal delay [7] could be a valuable issue for future research.

## Data Availability

Data sharing is not applicable to this article as no datasets were generated or analysed during the current study.
